# Supplementary material for: Intermittent brain network reconfigurations and the resistance to social media influence
Source: Netw Neurosci. 2022 Jul 1;6(3):870–96. doi: 10.1162/netn_a_00255 (PMC9810364; doi:10.1162/netn_a_00255)
Supplement: Supplementary file 1 [file netn-06-870-s001.pdf]

Lima Dias Pinto, I., Rungratsameetaweemana, N., Flaherty, K., Periyannan, A., Mghdadi, A., Richard, C., Berka, C., Bansal, K., Garcia, J. O. (2022). Supporting information for "Intermittent brain network reconfigurations and the resistance to social media influence." *Network Neuroscience*, 6(3): 870–896. https://doi.org/10.1162/netn_a_00255

Supplemental Material

EEG sensor position

The EEG recordings were obtained using the B-Alert R X24 wireless sensor headset (Advanced Brain Monitoring, Inc., Carlsbad, CA, United States), the system has 20 channels and the montage layout is presented in (Figure S1). The reference sensors were located behind each ear on the mastoid bone. The sample rate was 256 Hz with a high band pass at 0.1 Hz and a low band bass at 100 Hz.

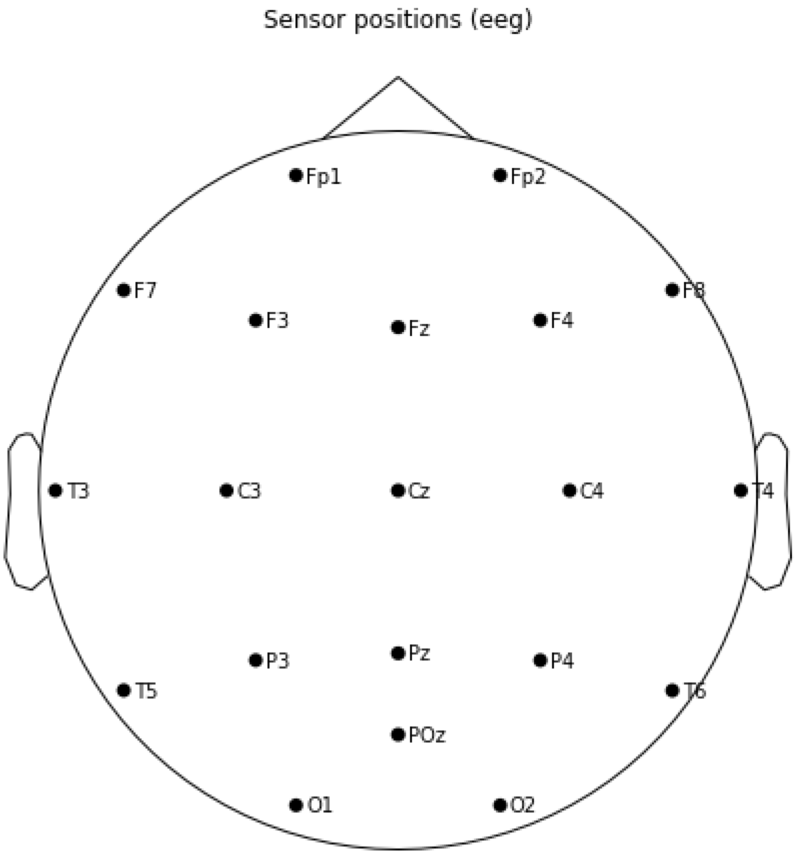

Figure S1: EEG sensor position. The topo plot shows the sensor montage. The EEG recording system model used was B-Alert R X24 wireless sensor headset (Advanced Brain Monitoring, Inc., Carlsbad, CA, United States) with 20 channels.

Node allegiance

To further understand the differences between those that did and did not change their opinion in terms of network reconfigurations, we explored how the network nodes, particularly the nodes that showed significant difference in flexibility between two groups, (significant nodes, Figure 2) changed their functional allegiances. Allegiance is defined as the fraction of the total time two nodes are in the same community. First, we calculated the allegiance metric between all the node pairs and then compared them between two groups. None of the node pairs (including or excluding the significant nodes) showed a significant difference between groups. Moreover, on average, some of the node-pairs showed higher allegiances for those without a change in opinion while some showed higher allegiances for those who changed their opinion. In Figure S2, we show average allegiance differences between two groups. Yellow entries in the matrices represent higher allegiances for those with no change. In topographical plots we show these differences only for the significant nodes.

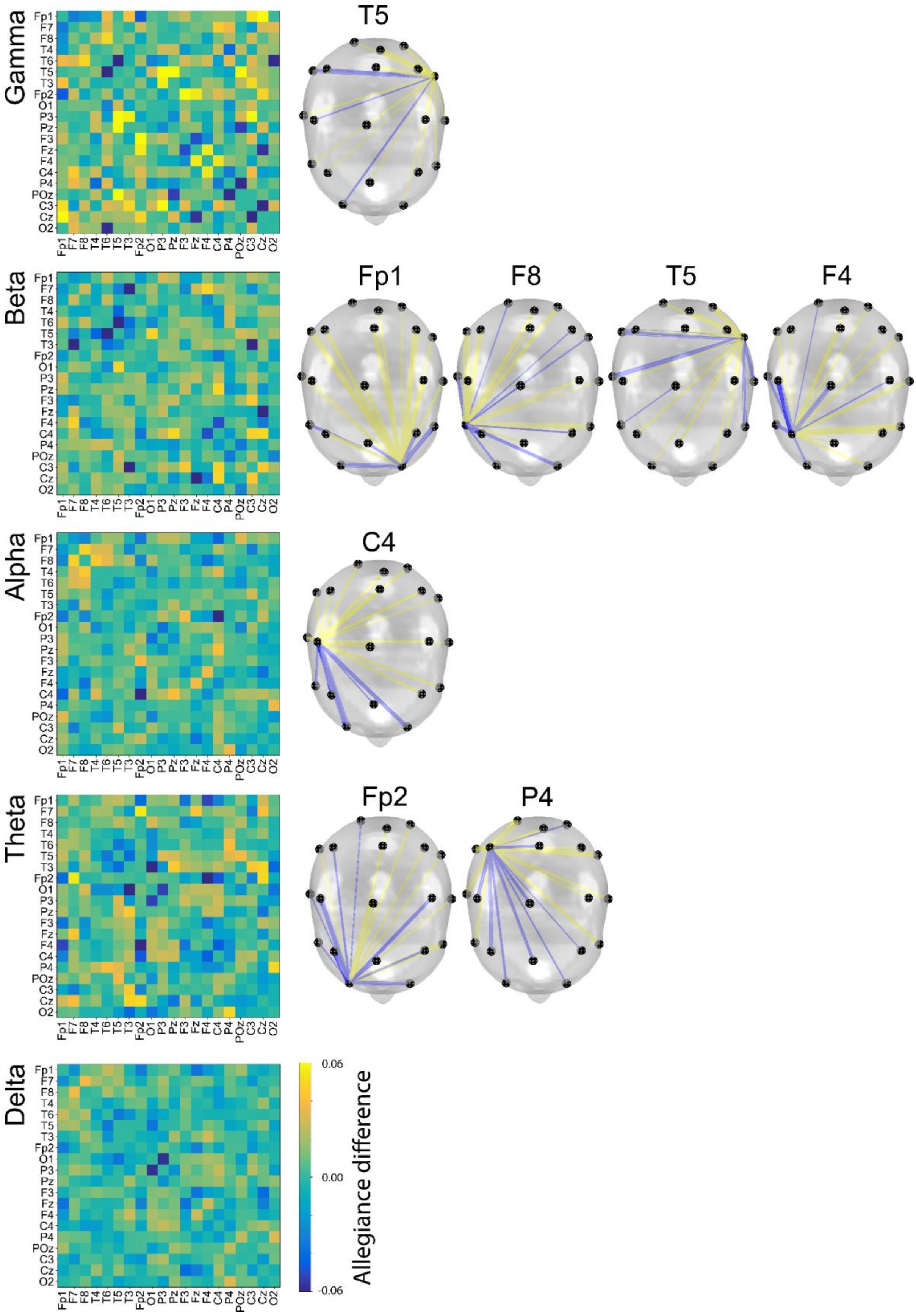

Gamma
T5
Beta
Fp1
F8
T5
F4
Alpha
C4
Theta
Fp2
P4
Delta
Fp1 F7 F8 T4 T6 T5 T3 Fp2 O1 P3 Pz F3 Fz F4 C4 P4 POz C3 Cz O2
0.06
0.00
-0.06
Allegiance difference

Figure S2: Node allegiance differences. The matrices show average allegiance differences between the two groups (no change and change). The topographic plots show the mean allegiance differences for sensors which showed significant flexibility changes between the two groups (as discussed in Figure 2). The links in yellow (blue) indicate a higher (lower) allegiance value for the no change(change) group.

Effects of temporal window sizes

To explore the effects of different temporal window sizes we compared the mean wPLI temporal coefficient of variation (CoV) for the opinionators with and without opinion change during the social media platform interaction, the results are summarized in Fig. S3. As a general effect of the increase in the window size we observe higher values and broader distributions of the mean temporal CoV. The differences between the two groups captured on the gamma band are present for temporal windows larger than 10s, indicating that smaller temporal windows do not capture the temporal scale of the dynamics that reflect the different processes occurring in the two groups.

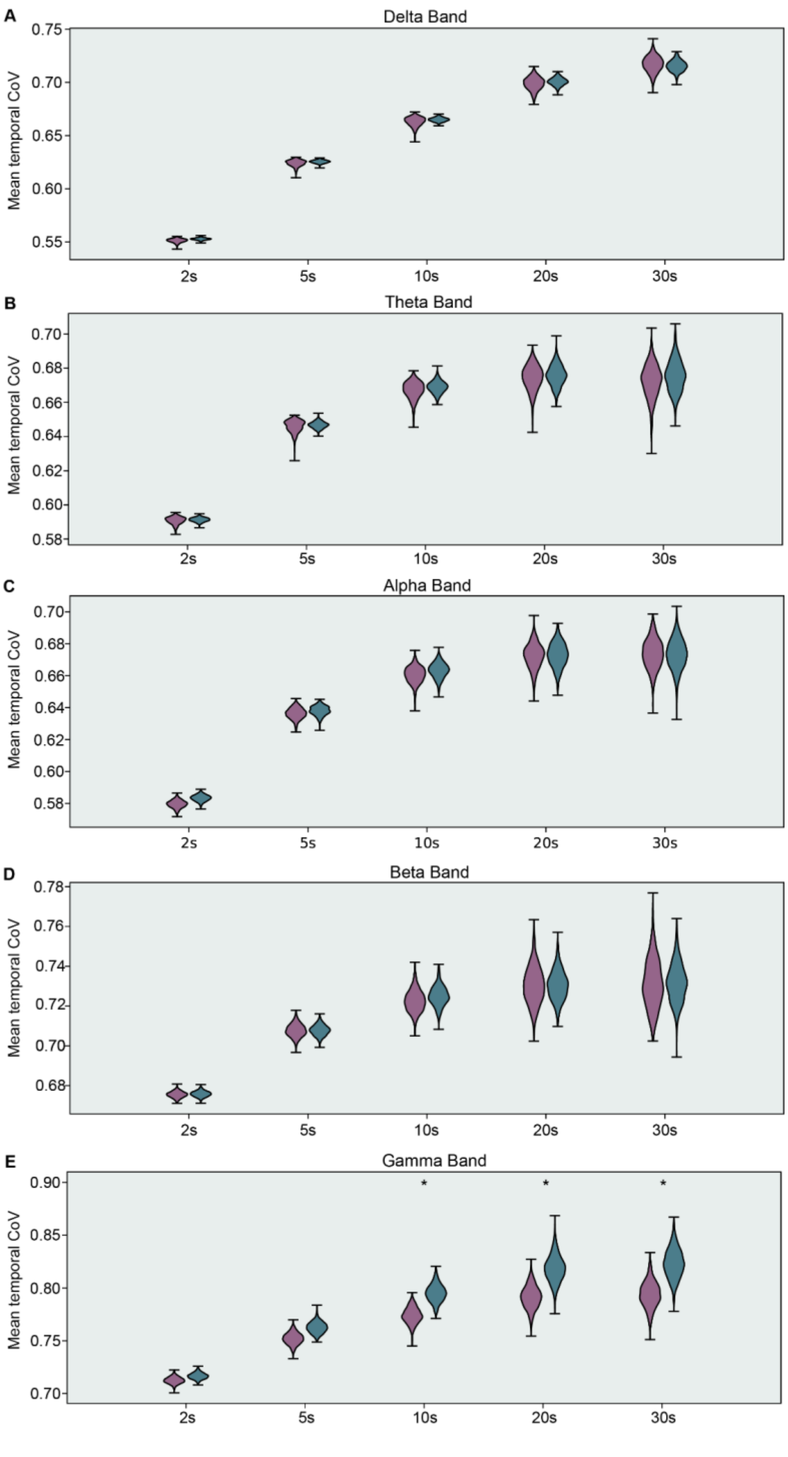

Fig S3: Effects of temporal window sizes. (A-E) Mean wPLI temporal CV for the those who do not change (purple) opinions and those who do (green) calculated for different temporal windows (2s, 5s, 10s, 20s, 30s) and bands (A) delta,(B) theta, (C) alpha, (D) beta, and (E) gamma. Statistical differences were estimated through a bootstrap procedure and significant results are denoted with an asterisk.

Opinion changes on Scenario 2 time in jail

A considerable amount of subjects (24 subjects) that changed opinions on scenario 2 changed only the time in jail. Since our group division on opinion change binarizes this response, a concern that might arise is if just a small change on the time in jail would classify a subject as having a change in opinion when there actually very little change (<1 year sentencing change, for example). To address this concern we plot the distribution of differences of time in jail between questionnaire 1 and 2, this is shown in Figure S4. All differences observed accounts for at least 10 years which is a substantial time and was subsequently considered an opinion change.

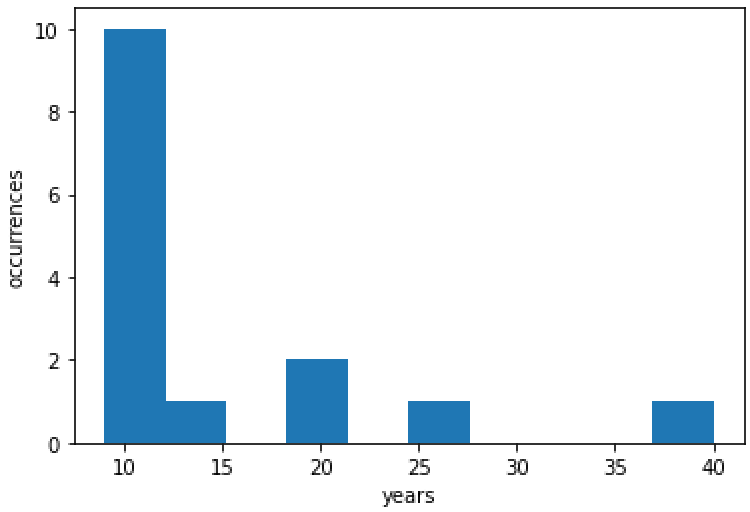

Figure S4: Histogram of time in jail changes in scenario 2. Occurrences of changes on time in jail question after interaction with the social media platform, the minimum change on time observed was 10 years. For this question, 9 subjects that changed their answers opted for either ‘death penalty’ or ‘life sentence’ as an answer in at least one of the two compared questionnaires.

Besides the changes in years in prison for a convicted murder, we also observed 9 subjects that showed opinion changes involving non-numerical values (death penalty and life sentence), the labels for those options were standardized to avoid identification of false differences due to typos and differences on capital letters.

Example articles presented to subjects:

As the posts presented to participants were inspired by real articles, below is a non-exhaustive list of some of content that was linked from the posts, from the travel scenario:

https://electrek.co/2019/04/09/paris-800-electric-buses/

https://www.audleytravel.com/indonesia/country-guides/sulawesi

https://www.bloomberg.com/news/articles/2019-04-06/yellow-vest-protesters-shift-focus-to-paris-business-district

https://www.npr.org/2018/09/28/652489085/strong-quake-hits-along-indonesias-western-sulawesi-island

https://www.express.co.uk/news/world/1110802/france-yellow-vest-protest-paris-riot-emmanuel-macron

https://www.nomadicmatt.com/travel-blogs/how-to-spend-5-days-in-paris/

https://www.youtube.com/watch?v=ErFP51JFUX8

https://www.nytimes.com/2019/03/16/world/europe/france-yellow-vests-protest.html

https://www.nytimes.com/2018/10/02/reader-center/donate-indonesia-tsunami-earthquake-victims.html

Comparison of link weights using wPLI and PLV

To disentangle the effects of PLV and wPLI, we first inspect the proportion of overlap at a variety of thresholds across wPLI and PLV. Figure S5 below visually depicts the proportion of edges that are overlapping after a binarization of the connectivity matrix for PLV and wPLI. Interestingly, within this dataset, at the extremes of connectivity, where t is the threshold of PLV and wPLI (t < 0.1 || t > 0.8), PLV and wPLI show very similar connectivity patterns; however, at the lower range (t > 0.15 && t < 0.6) there is substantial uniqueness of these connectivity patterns. We conclude that only the mid-range connectivity values have variable amounts of contribution from the common source problem, or volume conduction. In the figure below, we plot the mean, standard deviation, and coefficient of variation across time. For the mean, as the frequency increases, the overall mean decreases relative to the lower frequencies, suggesting that higher frequencies are less likely to be ambiguous in volume conduction effects or the “common sources problem”; however, the mean wPLI of the lower frequencies (e.g., delta, theta, alpha) is within the range of ambiguous sources.

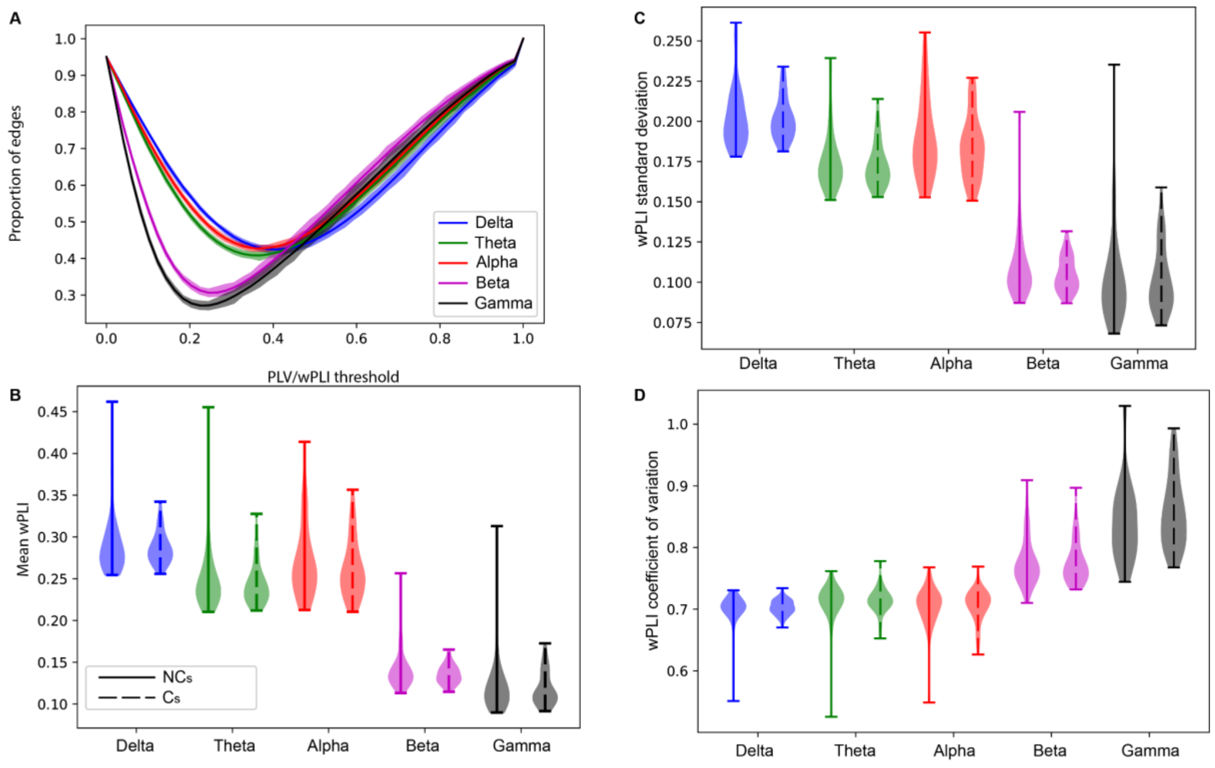

Figure S5: wPLI and PLV differences on the link weights. A Shows the comparison of wPLI and PLV edges present as we increase the threshold for considering an edge as present. B-D show, respectively the mean, standard deviation and coefficient of variation of the wPLI weights for both groups (opinion change and no change). Albeit the fact that the region of the minima is contained in the mean wPLI distribution range, the wPLI coefficient of variation shows that the distribution has a high variance.

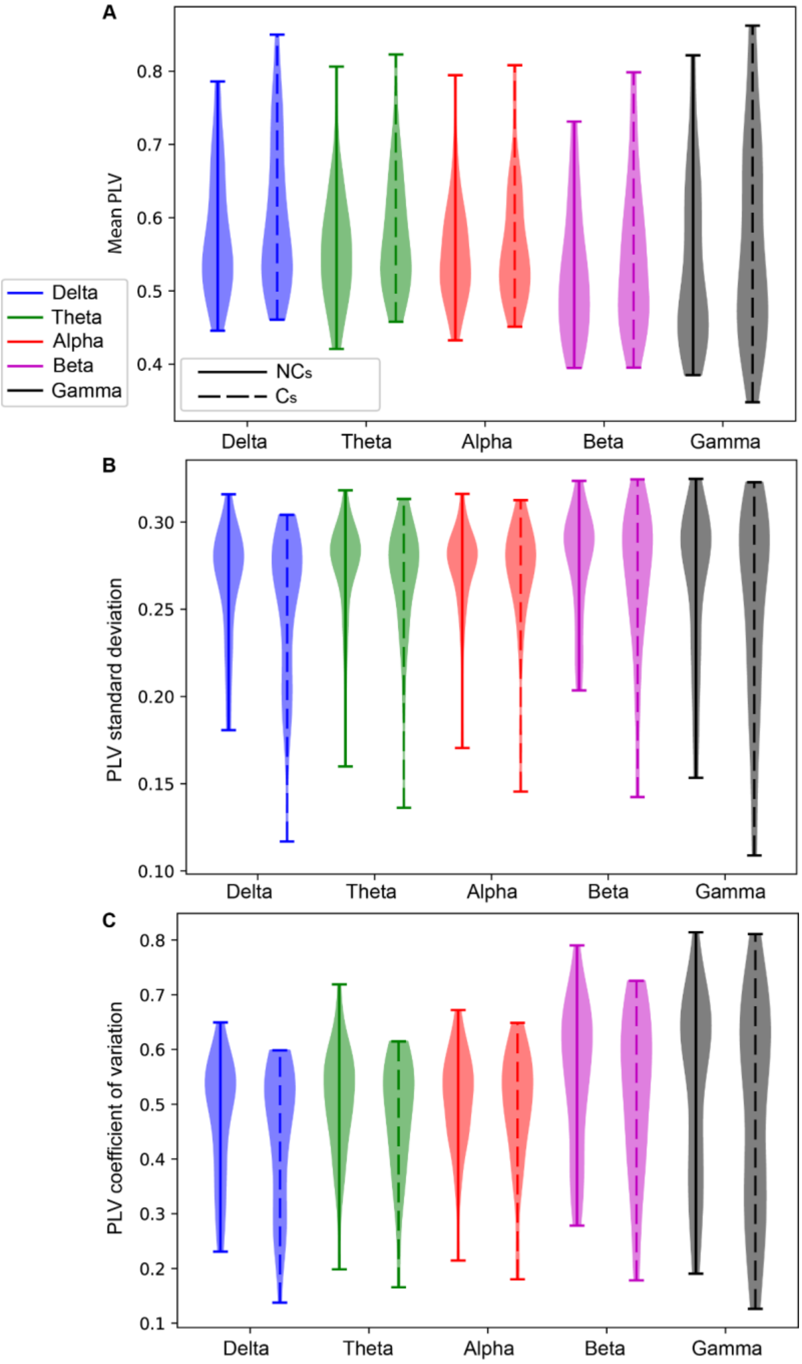

Figure S6: A-C show, respectively the mean, standard deviation and coefficient of variation of the PLV weights for both groups.

The statistics of the weight distributions calculated based on PLV are presented in Figure S6, the distributions cover a larger range of values when compared with wPLI with mean PLV distributed in a range of higher values than wPLI especially for the

higher frequency bands. From these two figures, considering the high overlap between metrics at the extreme values and the high variability in PLV that, it is unlikely that volume conduction significantly contributes to our results.

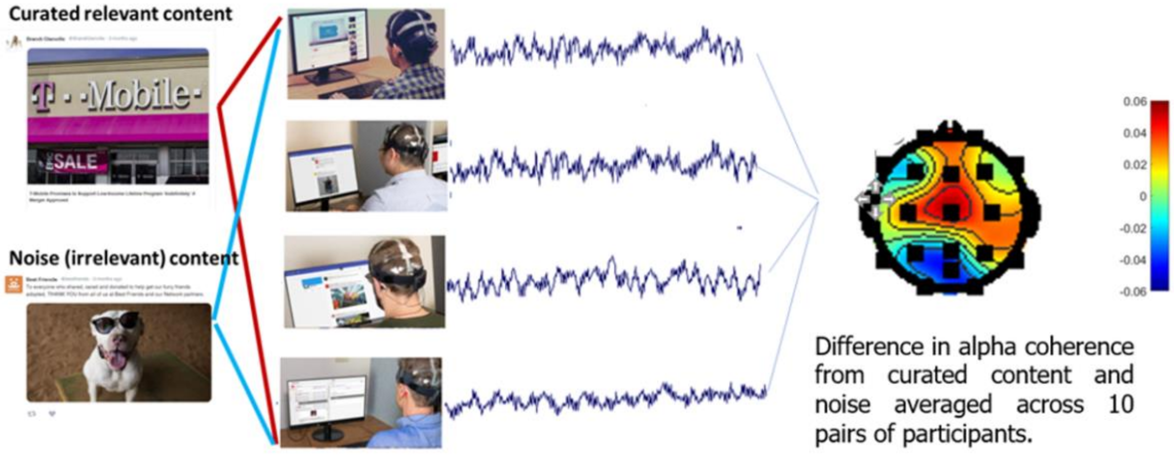

Figure S7: The platform and general setup. Subjects were presented with curated online content to elicit decision making processes (left) whilst instrumented with EEG (middle) and allowed to freely scroll through the content. High level alpha differences between rest and platform engagement are shown (right).
